# Gut microbiota-regulated tryptophan metabolism in breast cancer: mechanisms and therapeutic perspectives

**DOI:** 10.3389/fonc.2026.1765550

**Published:** 2026-02-25

**Authors:** Jiaxi Yan, Linfeng Qian, Shiqi Chen, Maryam Mohammed Abbas Karekad, Xuanwei Wu, Musheng Xu, Xiaohong Xu

**Affiliations:** 1 The First Clinical Medical College of Zhejiang Chinese Medical University, Hangzhou, China; 2 The International Education College of Zhejiang Chinese Medical University, Hangzhou, China; 3 Quzhou Traditional Chinese Medicine (TCM) Hospital at the Junction of Four Provinces Affiliated to Zhejiang Chinese Medical University, Quzhou, China; 4 The First Affiliated Hospital of Zhejiang Chinese Medical University (Zhejiang Provincial Hospital of Traditional Chinese Medicine), Hangzhou, China; 5 Bozhou District Hospital of Traditional Chinese Medicine, Zunyi, China; 6 The Affiliated Traditional Chinese Medicine Hospital of Shihezi University, Shihezi, China

**Keywords:** aryl hydrocarbon receptor, breast cancer, gut microbiota, kynurenine pathway, tryptophan metabolism, tumor immune microenvironment

## Abstract

Breast cancer remains the most commonly diagnosed cancer among women worldwide, and multiple studies now link its development and progression to disturbances in metabolic and immune regulation. Among these factors, the gut microbiota is increasingly recognized as a modulator of host physiology through its metabolism of dietary tryptophan (Trp). Here we focus on the current understanding of the microbial metabolism of Trp, which primarily generates bioactive metabolites through the kynurenine (Kyn) pathway and the indole pathway. These metabolites can serve as endogenous ligands, activating the aryl hydrocarbon receptor (AhR) signaling pathway. They can also promote tumor stem cell characteristics, epithelial-mesenchymal transition (EMT), and metastasis *via* serotonin receptors (such as HTR1B/1D, HTR2B). The activation of such pathways contributes to the remodeling of the tumor immune microenvironment, alters the functions of immune cells, and directly influences the proliferation, invasion, and metastatic behavior of breast cancer cells. By integrating findings from preclinical and clinical studies, this review organizes current evidence around the “gut microbiota-Trp metabolism-breast cancer” axis and discusses clinical implications and current limitations. Targeting this metabolic network may provide new opportunities for breast cancer prevention and therapeutic intervention.

## 1 Introduction

Breast cancer is the most frequently diagnosed malignancy among women worldwide, including in China (1, 2). In China, newly diagnosed cases are increasingly occurring in younger age groups, posing significant challenges for prevention and treatment (3). There is an urgent need to explore new therapeutic targets and strategies. The gut microbiota, often described as a “virtual organ” influences host physiology and disease pathogenesis through its metabolic activity and has been increasingly implicated in the development and progression of multiple cancers (4–6). Trp, an essential amino acid, serves as a precursor for signaling molecules, with its metabolic pathways playing central roles in immunoregulation and intercellular communication (7, 8).

Increasing evidences from both Chinese and international studies reveal that metabolites of Trp, such as Kyn and various indole derivatives, can, through signaling pathways such as the the AhR, subtly alter the immune microenvironment of breast tumors, influence the growth of cancer cells (9), and even interfere with the efficacy of chemotherapy and endocrine therapy (10–14). Trp metabolism derives not only from host cells, including both tumor cells and stromal cells, but is also shaped by the intestinal microbiota and microbiota localized within the tumor microenvironment (TME). Microorganisms contribute to the formation of a distinct metabolic profile either by directly degrading Trp or by modulating the activity of host metabolic enzymes (7, 15).

The relationship between microorganisms and host metabolism extends beyond simple coexistence. It is in fact governed by a dynamic interplay involving substrate competition, regulation through metabolite interactions (16), and shared signaling pathways (17).

For example, the gut microbiota can metabolize Trp into indole-derived compounds, thereby competing with the host for the same substrate. In contrast, host cells predominantly catabolize Trp through the Kyn pathway, mediated by indoleamine 2,3-dioxygenase 1 (IDO1), Indoleamine 2,3-dioxygenase 2 (IDO2), and tryptophan 2,3-dioxygenase (TDO). These enzymes are expressed across multiple cellular compartments, including tumor cells, antigen-presenting cells, and stromal components of the tumor microenvironment, where they contribute to the establishment of an immunosuppressive milieu (18, 19). Bacterial metabolites, such as indole-3-acetic acid (IAA), can inhibit host IDO1 activity through direct binding to its active site or by modulating upstream signaling pathways. This feedback mechanism ultimately suppresses Kyn production (20),moreover, host inflammatory signals (*e.g.*, IFN-γ) activate IDO1 in intestinal epithelial cells. This, in turn, alters the local Trp metabolic landscape, thereby shifting the gut microbiota profile and promoting the enrichment of tumor-promoting bacteria (21). Through this dynamic interaction, key downstream receptor signaling, such as the AhR pathway, is collectively regulated. Consequently, it shapes the tumor immune microenvironment, modulates cancer cell malignancy, and influences therapeutic responses, thereby contributing breast cancer progression (11).

Research into microbiota-metabolite-host interactions has progressed rapidly in recent years, providing important mechanistic insights and informing translational efforts (19, 22, 23). In particular, the establishment of the “microbiome-metabolite-cancer” axis has catalyzed the initiation of related clinical trials (24, 25). Despite these advances, substantial knowledge gaps remain regarding the specific roles of the microbiota-Trp axis in host physiology and disease. Key challenges include technical limitations in *in vivo* metabolite detection, context-dependent effects of distinct metabolic pathways, and barriers to clinical translation (22, 23). Clarifying these issues will be essential for determining how microbiota-driven Trp metabolism can be effectively exploited for therapeutic benefit.

With increasing recognition of metabolic reprogramming as a feature of tumor biology, attention has turned to the role of gut microbiota-regulated Trp metabolism in shaping breast cancer behavior at both local and systemic levels. This review examines current evidence on how microbiota-derived Trp metabolites influence breast cancer initiation, progression, and response to therapy, and discusses their potential implications for the development of preventive and therapeutic strategies targeting microbial metabolic pathways.

## 2 Trp metabolic pathways

**Figure 1:**
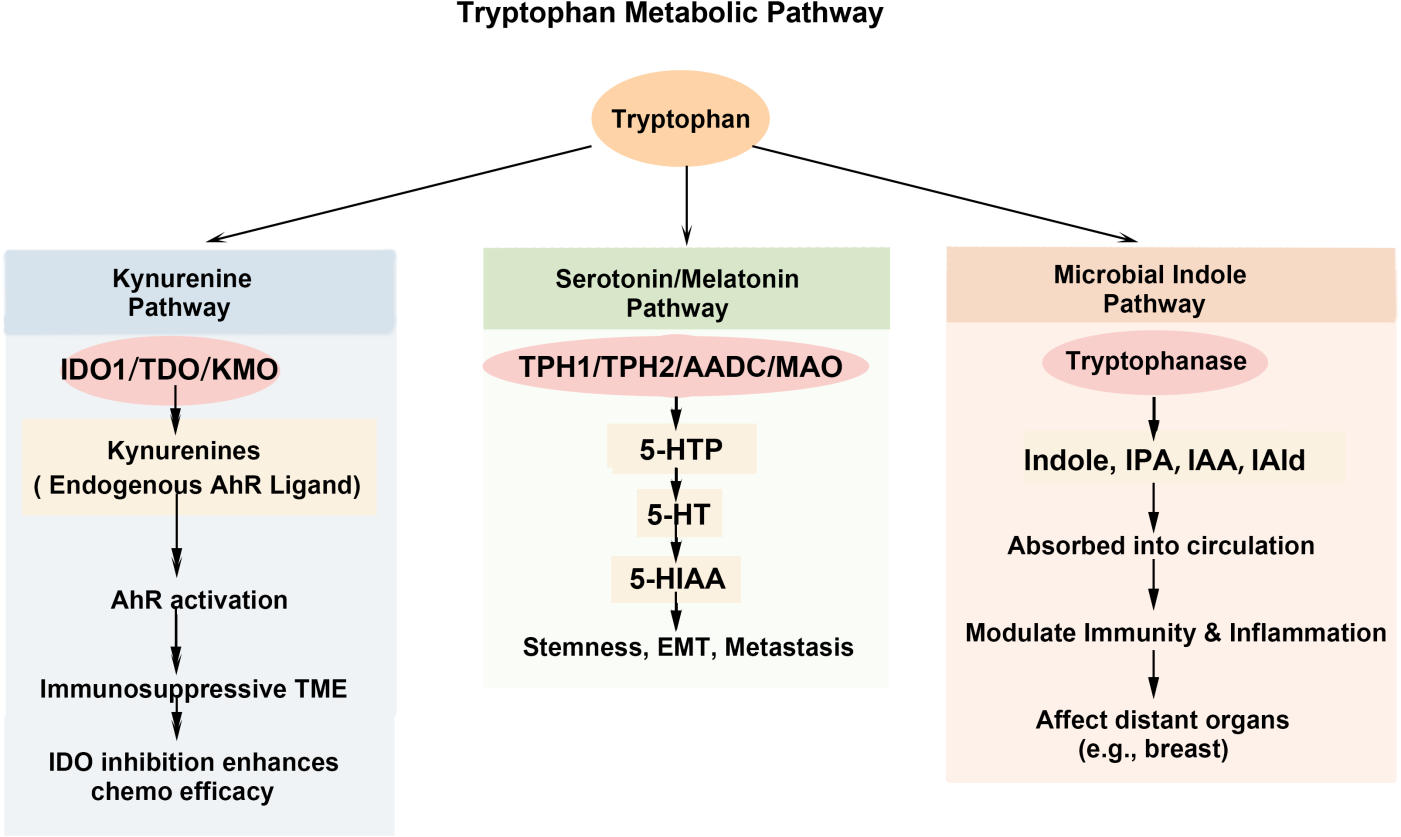
Major Trp metabolic pathways: Kyn pathway, microbial indole pathway, and serotonin/melatonin pathway.

Trp metabolism occurs primarily through three major pathways, which are summarized in **Figure 1**: the Kyn pathway, the serotonin/melatonin pathway, and the microbial indole pathway, each playing critical roles in maintaining homeostasis and influencing disease development (24).

### 2.1 The Kyn pathway: a core metabolic axis for immunomodulation

The Kyn pathway is the predominant route for Trp metabolism, accounting for approximately 95% of its catabolism (26). This pathway is governed by key rate-limiting enzymes, including IDO1/2, TDO, and kynurenine-3-monooxygenase (KMO) (27). Trp metabolism is initiated by oxidation and cleavage catalyzed by IDO1 or TDO, which gives rise to multiple downstream branches of the Kyn pathway. These reactions generate a range of metabolites, including 3-hydroxykynurenine, 3-hydroxyanthranilic acid, *N*-formyl Kyn, xanthurenic acid, kynurenic acid, anthranilic acid, and quinolinic acid. Collectively, these intermediates are referred to as Kyn pathway metabolites (28). The core function of this metabolic pathway lies in its potent immunomodulatory effects, which are mediated through two primary mechanisms. First, it rapidly depletes local Trp, leading to T-cell dysfunction and anergy. Second, its key metabolite, Kyn, serves as an endogenous ligand for the AhR. AhR activation drives the differentiation of Tregs and suppresses effector functions, thereby actively fostering an immunosuppressive TME (29–31).

### 2.2 The serotonin/melatonin pathway: a key neuro and peripheral regulatory route

The serotonin pathway is an essential biochemical process involved in regulating serotonin signaling, particularly within the central nervous system (CNS) (32, 33). Although this pathway accounts for only approximately 1-2% of total Trp metabolic flux (34–37), it exerts disproportionate biological effects. This apparent discrepancy reflects the high potency of serotonin as a receptor-mediated signaling molecule, whereby low ligand concentrations are sufficient to activate high-affinity receptors and elicit amplified downstream responses (38), including effects within immune cells and the tumor microenvironment (39).

The pathway begins with dietary Trp absorption into the circulation (40). In the brain, Trp is first hydroxylated by the rate-limiting enzyme Trp hydroxylase (TPH1 or TPH2) to form 5-hydroxytryptophan (5-HTP) (41). 5-HTP is then decarboxylated by aromatic L-amino acid decarboxylase (AADC) to generate serotonin (42, 43). Serotonin synthesis, transport, and turnover are tightly regulated by TPH isoenzymes, serotonin transporters, and multiple receptor subtypes. Newly synthesized serotonin may be released into the synaptic cleft to mediate signaling or stored in vesicles (44), while reuptake is controlled by the serotonin transporter (45). Ultimately, serotonin is oxidatively degraded by monoamine oxidase (MAO) on the outer mitochondrial membrane, producing its primary terminal metabolite, 5-hydroxyindoleacetic acid (5-HIAA) (46), which is subsequently excreted in the urine (47).

### 2.3 The microbial indole pathway: gut microbiota-driven diversified metabolism and systemic impact

The microbial indole pathway comprises multiple enzymatic reactions and represents a major route for Trp metabolism in the gut. In addition to decarboxylation reactions that generate tryptamine, gut microorganisms can convert Trp into a variety of indole derivatives through alternative biochemical processes, including deamination and side-chain cleavage. These pathways give rise to metabolites such as indole-3-propionic acid (IPA), indole-3-acetic acid (IAA), and indole-3-acetaldehyde (IAld) (48). These metabolites exhibit diverse biological activities: indole activates the pregnane X receptor (PXR) and AhR to enhance intestinal epithelial barrier function (49). IAId regulates IL-22 production, aiding mucosal homeostasis (50, 51). And IPA demonstrates potent antioxidant and anti-inflammatory properties (52). As natural AhR ligands, indole and its derivatives activate AhR signaling upon binding. This AhR-ligand complex then translocate to the nucleus and regulates the transcription of downstream genes involved in detoxification, immune regulation, and cellular development, playing a key role in modulating immune cell differentiation and overall immune responses (15).

### 2.4 Inter-pathway crosstalk: constituting a collaborative host-microbiota metabolic network

The microbial indole pathway and the host Kyn pathway do not operate in isolation; rather, they engage in continuous metabolic crosstalk. This creates an interdependent network governed by shared substrates and reciprocal feedback through interactive signaling (53). While intestinal microbiota transforms Trp into indole derivatives, host cells, specifically tumor cells and immune cells expressing IDO1/TDO, predominantly metabolize it through the IDO1/TDO-driven Kyn axis. Kyn pathway metabolites from these two arms bidirectionally regulate each other, demonstrating that systemic Trp metabolism is co-orchestrated by both microbial and host activities (18, 19). For example, bacterial-derived IAA can directly inhibit the enzymatic activity of host IDO1, thereby curtailing the downstream generation of the pro-cancerous metabolite Kyn (54). Conversely, under conditions of tumor or inflammation, host cytokines (*e.g.*, IFN-γ) perform a dual role: they upregulate IDO1 and concurrently remodel the intestinal microbiota. These changes reciprocally influence the composition of microbial indole metabolites, forming a dynamic feedback loop (21).

Acting as endogenous ligands, a range of metabolites—including indole, indole-3-acetaldehyde (IAld), and Kyn—can activate the AhR, which functions as a central integrator of downstream signaling. The biological consequences of AhR activation are highly context dependent and are influenced by multiple factors, including ligand identity and affinity, ligand concentration, target cell type, and the surrounding tissue microenvironment (55, 56). Within the tumor microenvironment (TME), AhR signaling is frequently associated with the establishment of immunosuppressive conditions, such as promotion of regulatory T cell (Treg) differentiation and inhibition of effector T cell activity (57, 58). However, under specific circumstances—particularly in the presence of high-affinity ligands—AhR activation has also been reported to exert direct tumor-inhibitory effects.

At the molecular level, AhR signaling follows a defined cascade. Microbiota-derived metabolites, together with certain host-derived ligands (including high-affinity indole derivatives and Kyn), bind to and activate AhR (59, 60). Upon activation, AhR translocates to the nucleus and heterodimerizes with the AhR nuclear translocator (ARNT), enabling the complex to bind xenobiotic response elements (XREs) within target gene promoters. The resulting transcriptional programs regulate genes involved in cell cycle control, apoptosis, metabolic adaptation, maintenance of stemness, and cell adhesion and migration. Through these mechanisms, metabolite-driven AhR signaling can directly influence key malignant phenotypes, including tumor cell proliferation, metabolic reprogramming, and metastatic potential (61, 62).

Thus, the metabolomic network orchestrated by the host and gut microbiota drives breast cancer progression through multifaceted mechanisms. These include remodeling the tumor immune microenvironment, directly interfering with cancer cell behavior, and perturbing systemic inflammation and metabolic homeostasis. To elucidate how gut microbiota exert remote metabolic control, research must shift focus from isolated pathways to the integrated network itself. This systemic understanding is essential for developing targeted therapies, such as precise dietary regimens, probiotic interventions, or small-molecule drugs, aimed at this critical network.

## 3 The interaction between gut microbiota and Trp metabolism affects immune regulation

The crosstalk between gut microbiota and host Trp metabolism is a central mechanism in maintaining systemic homeostasis, involving multiple levels of regulation such as substrate competition, metabolite signaling, and systemic immunomodulation (63–65).

### 3.1 The common interaction between the host and microorganisms in the metabolism of Trp

Gut microbiota directly competes with the host for dietary Trp by expressing microbial Trp-degrading enzymes, such as tryptophanase and bacterial-type IDO-like enzymes. For instance, *Escherichia coli* converts Trp into indole (66), while *Clostridium* sp*orogenes* can produce beneficial metabolites such as IPA (52, 63, 67, 68). It is important to distinguish host and microbial Trp metabolism. In host cells, Trp is predominantly catabolized through the Kyn pathway mediated by IDO1/IDO2 and TDO2, whereas gut microorganisms utilize distinct and independent catabolic pathways. These host and microbial systems differ fundamentally in their enzymatic origins and metabolic routes. Microbial indole metabolites compete with host cells, particularly tumor cells, for the essential amino acid Trp through both direct and indirect mechanisms. Directly, microorganisms consume and utilize Trp as a metabolic substrate; indirectly, microbial metabolites can modulate the expression or activity of host enzymes such as IDO1, either enhancing or suppressing Trp catabolism. In parallel, microbial indole compounds and their downstream metabolites can enter the systemic circulation, where they influence immune responses and inflammatory states. Through these mechanisms, microbiota-derived indole metabolism can affect physiological and pathological processes in distant organs, including breast tissue (63, 65, 69), hereby forming a metabolic link between the gut microbiota and systemic diseases such as breast cancer.

### 3.2 Regulation of intestinal barrier integrity by microbial metabolites

Indole and its derivatives act as endogenous ligands of the AhR, activating AhR signaling in epithelial cells and promoting the expression of tight junction proteins (*e.g.*, occludin, claudin-1), thereby enhancing gut barrier function (51), inhibiting the Nuclear Factor kappa-light-chain-enhancer of activated B cells (NF-κB) pathway and NOD-like receptor protein 3 (NLRP3) inflammasome activation, and reducing the release of pro-inflammatory cytokines such as TNF-α and IL-6 (70, 71). Limosilactobacillus r*euteri* alleviated inflammation by producing Indole-3-lactic acid (ILA) and IPA that activate AhR, thereby inhibiting Th2 cytokines (IL-4, IL-5) and thymic stromal lymphopoietin (TSLP) expression (50, 72, 73). These data support the pivotal role of the microbiome-AhR axis as an interface that couples microbial metabolic output to the regulation of epithelial integrity and immune responses.

### 3.3 Systemic immune regulation and its effects on peripheral organs

Trp metabolism acts as a critical link between gut microbiota and host health. Its dysregulation is implicated in various pathologies, including cancer, neurodegenerative diseases, and autoimmune disorders (48). As the primary site of Trp metabolism, the liver is significantly influenced by gut-derived microbial metabolites (74). Under homeostatic conditions, beneficial gut bacteria convert Trp into indole derivatives, such as IPA. These metabolites reach the liver *via* the portal vein and function as ligands for the AhR. AhR activation exerts immunostimulatory effects, including the promotion of Th17 cell differentiation, which supports intestinal barrier integrity and host defense (7, 75).

During dysbiosis—for example, during *Enterococcus* overgrowth—the Trp metabolic pathway alters. Production of beneficial metabolites like IPA declines, while harmful products such as indoxyl sulfate accumulate. This shift promotes hepatic inflammation and fibrosis (69, 76–79). Furthermore, broad-spectrum antibiotic treatment can remodel the gut microbiota, increasing the diversion of Trp into the Kyn pathway (80–82).

Notably, the immunomodulatory role of AhR is highly context-dependent and exhibit marked cell-type specificity. The outcomes of AhR signaling depend not only on the ligand type, local microenvironment, and host physiological status (56), but are also strongly influenced by cell type (*e.g.*, tumor cells, T cells, myeloid cells), the nature and affinity of ligands, the local cytokine milieu, and the stage of disease (55, 59, 83–85). In chronic inflammatory settings (*e.g.*, autoimmune diseases), the Trp metabolite Kyn activates AhR to drive Tregs differentiation and induce a tolerogenic phenotype in dendritic cells (DCs), thereby promoting immunosuppression and anti-inflammatory responses (56, 75, 86, 87). Similarly, within the tumor microenvironment, AhR signaling may enhance immunosuppression under certain conditions, such as Treg polarization or the induction of tolerogenic DC phenotypes (85, 88). Conversely, in chronically stimulated CD8^+^ T cells, the same signaling axis can lead to effector dysfunction or exhaustion, with specific outcomes varying depending on the experimental model and tissue site. These effects are not mutually exclusive and can coexist within the same TME, collectively shaping the overall immune landscape (89). Under homeostasis, microbiota-derived indole compounds activate AhR to enhance Th17 responses and support immune surveillance (88).

This bidirectional regulation indicates that microbial metabolites not only directly affect liver inflammation and fibrosis through pathways involving AhR, but also modulate AhR activity in hepatocytes and the expression of cytochrome P450 enzymes. Consequently, they influence drug metabolism and toxicity (90–92). Thus, the gut microbiota exerts systemic effects beyond the intestine, shaping metabolic and immune responses across multiple organs.

### 3.4 Impact on the tumor immune microenvironment

Within the TME, microbial and host-derived metabolites activate the AhR signaling pathway to regulate immune cell differentiation and function. AhR activation drives CD8+ T cell exhaustion and suppresses antitumor immunity, thereby facilitating immune escape and tumor progression. Specifically, tumor-initiating cells can exploit the Kyn-AhR axis to upregulate PD-1 expression on CD8+ T cells, thereby amplifying immunosuppression and protumor effects (30). Thus, by modulating the production and signaling of Trp metabolites, the gut microbiota directly influences systemic immune balance and shapes antitumor immune responses.

**Figure 2:**
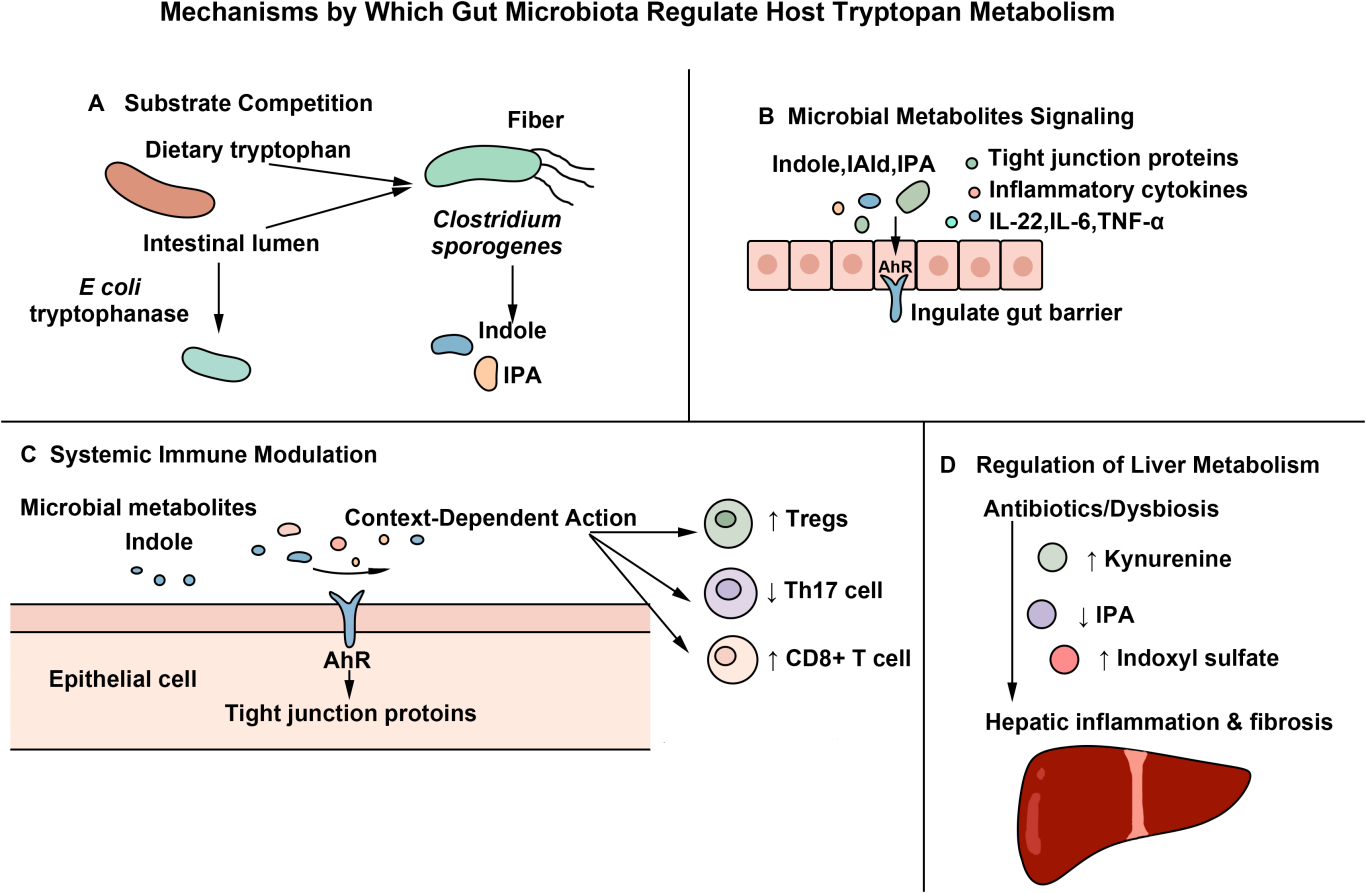
Mechanisms by which gut microbiota regulate host tryptophan metabolism. **(A)** Substrate Competition and Metabolic Diversion: Gut microbiota competes with the host for dietary Trp, converting it into various metabolites. This process can be modulated by diet to promote the production of beneficial anti-inflammatory metabolites; **(B)** Local Signaling and Homeostasis: Trp metabolites derived from the microbiota activate the AhR signaling pathway. This enhances intestinal barrier function and exerts local anti-inflammatory effects; **(C)** Remote influence, regulation of immunity: These metabolites regulate the functions of immune cells through pathways such as AhR, affecting the overall immune balance and antitumor immunity. Their effects are environment-dependent; **(D)** Remote influence, acting on the liver: The metabolites of the microbiota affect the liver through the bloodstream, and are associated with liver inflammation, fibrosis, and drug metabolism.

In summary, a deeper understanding of these pathways (especially their tissue-specific effects) is crucial for advancing microbiome-based therapeutic strategies targeting the “ microbiota-metabolism-immune” axis. **Figure 2** summarizes the overview of how the gut microbiota regulates the host’s availability of Trp, signaling, and immune response through the aforementioned coordinated mechanisms.

## 4 The mechanism by which Trp metabolism promotes the progression of breast cancer

**Figure 3:**
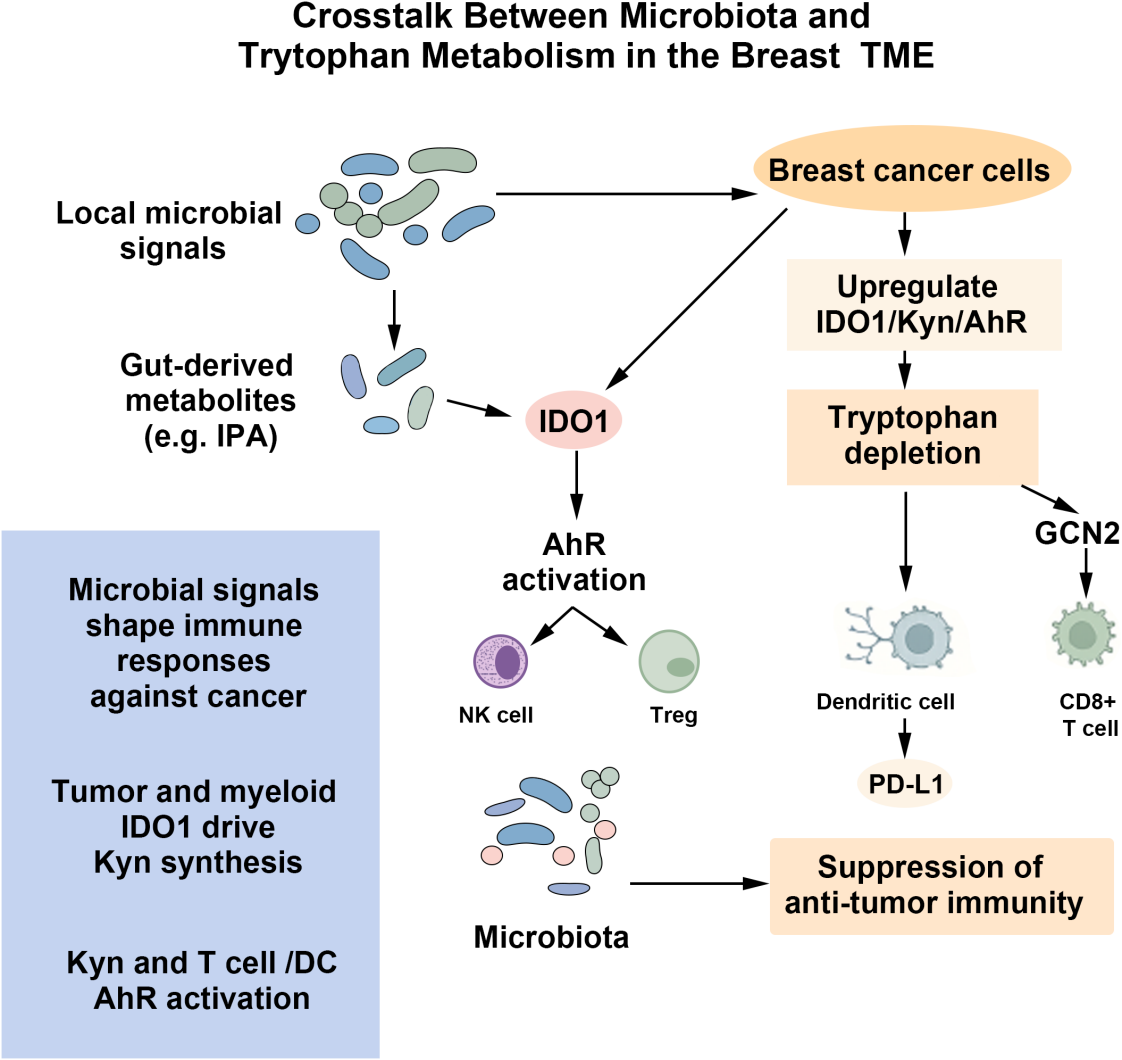
Crosstalk between microbiota and Trp metabolism in the TME. Microbiota-regulated Trp metabolism promotes breast cancer progression through dual mechanisms. In the TME, IDO1 upregulation in cancer cells depletes Trp and accumulates Kyn. Trp starvation activates GCN2 in CD8^+^ T cells, impairing their function. Kyn activates the AhR in immune cells, driving PD-L1 expression and Tregs differentiation to suppress antitumor immunity. AhR signaling also directly enhances tumor cell growth. These pathways collectively establish an immunosuppressive microenvironment that enables immune escape and tumor progression.

The regulation of Trp metabolism by the gut microbiota significantly influences the occurrence and development of breast cancer through multiple mechanisms. Its core functions can mainly be divided into the remodeling of the tumor immune microenvironment and the direct driving of breast cancer cells, and it forms a complex regulatory network by coordinating different receptor signaling pathways. **Figure 3** illustrates how microbial metabolites and host-derived Kyn converge on IDO1-AhR signaling to remodel the tumor immune microenvironment and promote immune evasion.

### 4.1 Reconfiguring the immunosuppressive TME to facilitate immune escape

Trp metabolism is a key interface in shaping the immunosuppressive TME. In the TME of breast cancer, the uptake of Trp mainly relies on L-type amino acid transporter 1 (LAT1), which is upregulated in both primary and metastatic breast cancer lesions and is associated with poor prognosis (93–95). After entering the cells, Trp is mainly oxidized and degraded through the Kyn pathway. The rate-limiting enzyme of this process, IDO, is highly expressed in various tumors, including breast cancer, and its level is closely related to tumor growth, metastasis, and poor prognosis (13, 14, 96). This metabolic reprogramming directly leads to Trp depletion and accumulation of Kyn in the TME, which in turn suppresses antitumor immunity through multiple mechanisms: Trp depletion activates the General Control Nonderepressible 2 (GCN2) kinase pathway in CD8^+^ T cells, causing cell cycle arrest and functional exhaustion (97); while accumulated Kyn can induce antigen-presenting cells such as DCs to express immune checkpoint molecules like PD-L1 and promote the differentiation of Tregs, thereby comprehensively suppressing the immune response (30, 98). The activation of AhR in T cells and dendritic cells not only promotes the differentiation of Tregs, but also enhances their inhibitory ability and phenotypic stability after maturation, thereby more effectively suppressing the activity of effector T cells and natural killer (NK) cells (98). IPA has been demonstrated to enhance the antitumor function of CD8+ T cells by promoting histone acetylation and increasing the chromatin accessibility at effector gene loci, thereby facilitating epigenetic reprogramming (99). This complex interaction reveals a crucial “gut-tumor” metabolic axis that affects immune evasion (99, 100).

### 4.2 Directly driving breast cancer progression through the AhR signaling pathway

The impact of Trp metabolites on breast cancer extends beyond shaping the immunosuppressive microenvironment. As the common receptor for various Trp metabolites such as Kyn, the AhR signaling pathway plays a direct and multifaceted driving role in breast cancer cells (101, 102). Whether it is indole derivatives from microorganisms or Kyn from the host, they can activate AhR, thereby promoting the proliferation, EMT (103), and maintenance of cancer stem cell characteristics of breast cancer cells (50, 104). Mechanistically, after AhR activation, it translocates to the nucleus and forms a heterodimer with ARNT, regulating the expression of downstream genes such as *CYP1A1* and *CYP1B1 (*105, 106). This activation has subtype-specific effects. For example, in the triple-negative breast cancer (TNBC) model, the AhR signal enhances the invasiveness of tumor cells through the TWIST1/SNAIL1 axis. In estrogen receptor-positive (ER+) breast cancer, the crosstalk between AhR and ER affects the sensitivity to endocrine therapy (such as tamoxifen) (107).

It is worth noting that the role of the AhR signal in breast cancer is highly dependent on the environment, exhibiting both tumor-promoting and potential tumor-suppressing properties (108, 109). This effect depends on the type of ligand, activation level, duration, and cellular background. Although the studies and most evidence from TMEs support its oncogenic role, certain specific ligands (such as some dietary ligands) or in the context of detoxification of exogenous toxins, the activation of AhR may also trigger cell cycle arrest or apoptosis programs, demonstrating tumor-suppressive potential (110). In the pathological context of breast cancer, especially in the microenvironment dominated by the continuous activation of the IDO/Kyn axis, the AhR signal typically tends to promote tumor progression and immune escape (111). The comprehensive understanding of this complexity is crucial for the design of therapeutic strategies targeting AhR.

### 4.3 Forming regulatory networks through the synergy of multiple receptor signaling pathways

The influence of the Trp metabolic network is also amplified by the simultaneous activation of different receptor signaling pathways. Besides AhR, metabolites of the Kyn pathway can also transmit signals through G protein-coupled receptor 35 (GPR35) and other receptors, further regulating cellular functions. This multi-receptor synergy is particularly significant in ER+ breast cancer, forming a close interaction between the microbiota-regulated Trp metabolism and estrogen signaling networks. On one hand, the intestinal microbiota expressing β-glucuronidase can hydrolyze estrogen-glucuronide conjugates, promoting the intestinal-portal circulation reabsorption of free estrogen, thereby increasing estrogen exposure in breast tissues (112, 113). On the other hand, Trp metabolism and estrogen signaling can directly interfere with each other through multiple mechanisms: AhR can form a protein complex with ERα, mutually regulating each other’s transcriptional activity (114); Trp metabolic enzyme CYP1B1 participates in the hydroxylation of estrogen, generating 4-hydroxy estrogen metabolites with genotoxicity (115); Kyn pathway metabolites can also regulate the phosphorylation state of ER through activating the GPR35 receptor. Preclinical studies have shown that dietary supplementation of Trp can weaken the estrogen sensitivity of breast tumors by altering the microbiota composition and reducing the activity of β-glucuronidase, providing a new idea for dietary intervention in ER+ breast cancer (116).

### 4.4 Subtype-specificity and therapeutic prospects

The above mechanisms collectively form a complex network of the microbiota-Trp axis influencing the pathogenesis of breast cancer. It is notable that different breast cancer subtypes may exhibit distinct metabolic dependencies: for example, the activation of the IDO1/Kyn/AhR axis is usually more significant in TNBC (28), while the interaction between Trp metabolism and estrogen signaling is more prominent in ER+ breast cancer (52). Identifying these subtype-specific metabolic features is crucial for designing precise treatment strategies, such as combining IDO inhibitors with immune checkpoint blockers for TNBC (116, 117), or using probiotics/dietary intervention to regulate the activity of microbial β-glucuronidase as an adjunctive treatment for ER+ breast cancer endocrine therapy (118). Future research should focus on elucidating the heterogeneity of the Trp metabolic network in different breast cancer subtypes and individual patients, and on evaluating the clinical translational potential of microbiota-targeted intervention measures based on this.

## 5 Targeting the gut microbiota-Trp-breast cancer axis: breast cancer treatment and transformation

The gut microbiota regulates Trp metabolism to form the “microbiota-Trp-breast cancer axis”, which integrates metabolic, immune and hormonal signals and affects the occurrence and development of breast cancer (7, 19, 119). The mechanism mainly includes microbial-derived indole substances and host metabolites such as Kyn, which directly regulate the malignant behavior of tumor cells and reshape the tumor immune microenvironment by activating receptors such as AhR; in ER+ breast cancer, it can also interact with estrogen signals to exacerbate progression and treatment resistance. This axis provides new potential targets and transformation directions for the precise treatment of breast cancer (105, 112). **Table 1** summarizes representative microbial species, their key metabolites, and associated roles in breast cancer, demonstrating the functional diversity within this regulatory network.

**Table 1:** Representative microbial species, their metabolites, and roles in breast cancer.

Microbial species/cells	Major metabolites	Receptor/target	Biological effects	Mechanistic outcome
*Lactobacillus* spp. *Bifidobacterium* spp.	ILA IPA	AhR	Enhanced intestinal barrier integrity Modulated immune homeostasis, suppression of pro-inflammatory cytokines	Antitumor
*Limosilactobacillus reuteri*	ILA IPA	AhR	Activation of AhR, inhibition of Th2 cytokines (*e.g.*, IL-4, IL-5) Attenuation of inflammation	Antitumor
*Clostridium* sp*orogenes*	IPA	AhR	Potent antioxidant and anti-inflammatory effects Systemic immunomodulation	Antitumor
*Escherichia coli*	Indole Indoxyl Sulfate	AhR PXR	Low concentrations of indole enhance intestinal barrier Indoxyl sulfate promotes inflammation and fibrosis	Pro-tumor (context- dependent)
*Bacteroides* spp.	(*Via* β-glucuronidase) Liberated Estrogen	ER	Increased systemic free estrogen levels Promotion of ER^+^ breast cancer cell proliferation	Pro-tumor (in ER^+^ breast cancer)
Tumor/Immune Cells	Kyn	AhR	Promotion of Tregs differentiation Suppression of CD8^+^ T cell and NK cell function	Pro-tumor (immunosuppressive)
Breast Cancer Cells	serotonin	Serotonin receptors (*e.g.*, HTR1B/1D, HTR2B)	Autocrine activation of the YAP signaling pathway Promotion of cancer stemness, EMT, and metastasis	Pro-tumor

The overall impact (pro-tumor or antitumor) of a metabolite is highly context-dependent, influenced by factors such as concentration, local microenvironment, and cancer subtype.

Clinically, this metabolic axis offers a promising set of biomarkers for the diagnosis and prognosis of breast cancer. Studies have shown that combining the detection of reduced fecal levels of indole-producing bacteria (*e.g.*, *Lactobacillus* and *Bifidobacterium*) with an elevated serum Kyn/Trp ratio can improve the diagnostic sensitivity for early-stage breast cancer (120). An elevated serum Kyn/Trp ratio (*e.g.*, >35 nmol/μmol) serves as an independent prognostic factor in ER+ breast cancer, associated with up to a 3.2-fold increased risk of disease progression (62).Additionally, baseline urinary levels of microbial indole derivatives, such as IAA, correlate with the rate of pathological complete response following neoadjuvant chemotherapy, suggesting their potential as predictors of treatment efficacy (121). These findings indicate that combining microbial and metabolic markers could enhance early detection and refine risk stratification in breast cancer (122).

**Figure 4:**
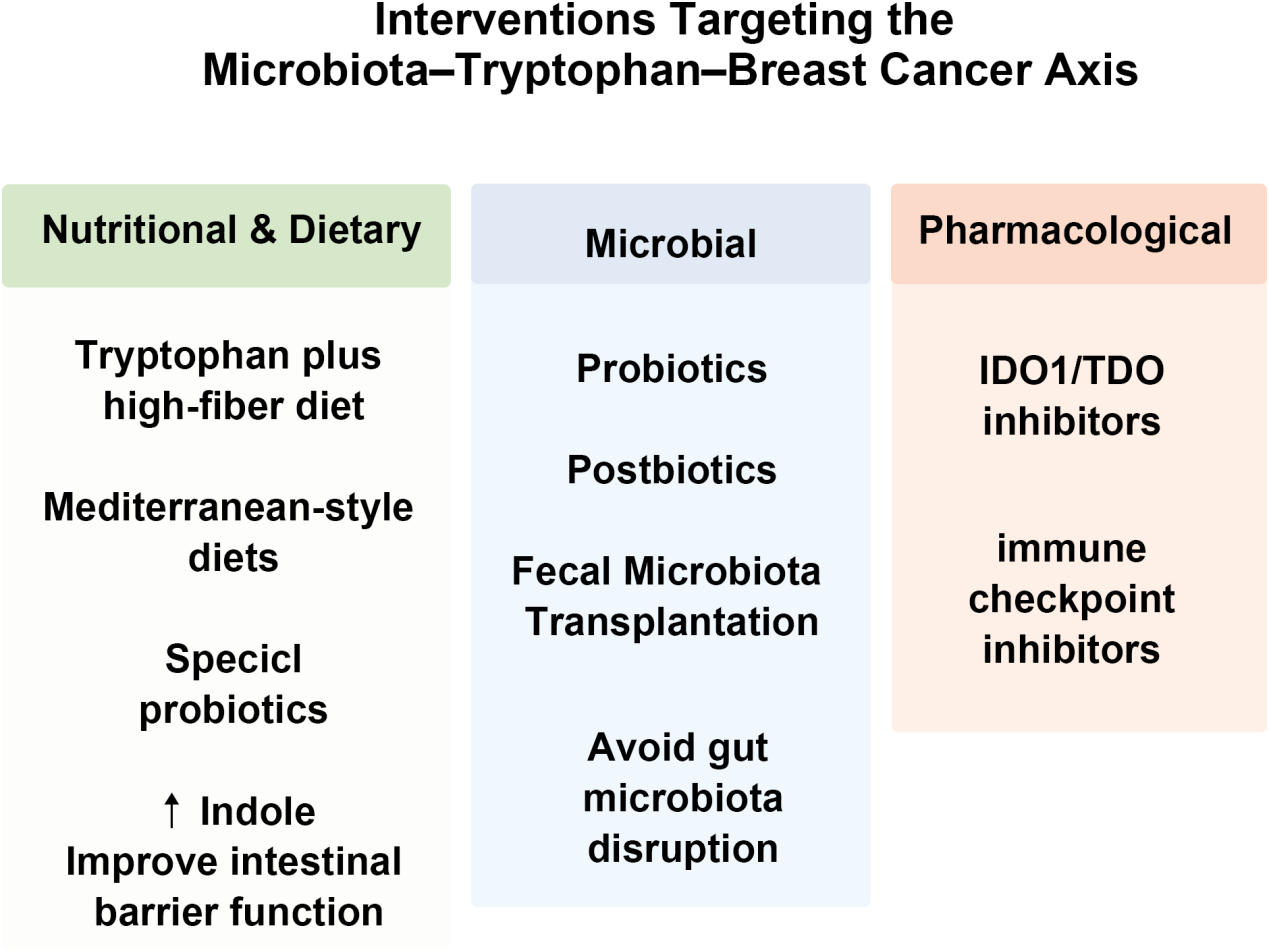
Interventions targeting the microbiota-Trp-breast cancer axis. Through dietary modification, microbiota-based interventions, and targeted pharmacological approaches, modulation of the microbial Trp metabolic axis may represent an emerging component of precision medicine strategies for breast cancer.

Based on the pathological mechanisms and clinical value revealed by these biomarkers, intervention strategies targeting the “microbiota-Trp-breast cancer axis” are being actively developed, aiming to translate the understanding of its mechanism into clinical treatment. As our understanding of the microbiome-Trp-breast cancer axis advances, researchers have begun exploring novel therapeutic strategies aimed at reprogramming this metabolic network. These approaches fall into three main categories: nutritional intervention, microbial modulation, and pharmacological targeting (7, 123, 124). Key nutritional, microbial, and pharmacological interventions targeting this metabolic axis are summarized in **Figure 4**.

Dietary intervention represents a foundational strategy. Increasing Trp intake alongside a high-fiber diet can promote beneficial gut bacteria, steering their metabolism toward producing advantageous indole derivatives (*e.g.*, IPA and ILA), while concurrently suppressing the generation of harmful metabolites (19, 63, 125). Clinical trials have shown that a Mediterranean diet rich in Trp and fiber can increase the level of indole in the feces of breast cancer patients and improve the intestinal barrier function (126).

Microbial intervention represents another key approach, utilizing specific probiotics such as *Lactobacillus reuteri* and *Bifidobacterium longum* to restore microbial balance and boost the production of beneficial metabolites. More targeted strategies are also being developed, including the use of “postbiotics”, purified bacterial metabolites, as a direct therapeutic modality. For example, oral administration of IPA has demonstrated significant antitumor effects in preclinical models (78). For patients with severe microbial dysbiosis-such as those receiving immunotherapy-fecal microbiota transplantation (FMT) can help restore microbial diversity and improve Trp metabolism, thereby enhancing the response to immune checkpoint inhibitors like PD-1 blockers (127–129).

Although the first-generation selective IDO1 inhibitors (*e.g.*, epacadostat) failed to improve patient prognosis in key phase III clinical trials (116), this suggests that due to the overexpression and activity of IDO1 in the TME being difficult to be completely and continuously inhibited by pharmacological means (130), and the existence of compensatory mechanisms such as enzymes like TDO in the Trp metabolic pathway (29, 131), the efficacy of single IDO1 inhibitors is limited. However, drug development targeting the complete immune metabolic axis of IDO1/TDO-Kyn-AhR is still actively ongoing, with strategies shifting towards the development of dual inhibitors or AhR antagonists (132). Novel approaches such as dual IDO1/TDO inhibitors and selective AhR modulators, particularly in combination with immune checkpoint inhibitors, have shown promising potential (10). Furthermore, the judicious use of antibiotics during therapy is critical, as broad-spectrum antibiotics can disrupt both the microbiota and Trp metabolism, an effect linked to reduced survival rates in breast cancer patients (133). This underscores the importance of preserving microbial ecology throughout the course of treatment.

Although the aforementioned intervention strategies demonstrate potential for transformation, the clinical application still faces systematic challenges. The complexity and individual variability of both the microbiome and host metabolism necessitate personalized therapeutic regimens (134, 135), and the dynamic nature of the microbiota requires continuous monitoring (21). Future large-scale randomized controlled trials will be essential to establish causality and identify optimal intervention strategies (136). Furthermore, the establishment of standardized protocols for microbiome and metabolome analysis is a prerequisite for robust clinical application (137, 138). As these hurdles are progressively addressed, strategies targeting the “microbiota-Trp-breast cancer axis” are anticipated to become a vital component of the precision medicine framework for breast cancer.

## 6 Conclusions

This review highlights the role of the gut microbiota-Trp metabolism axis in the regulation of breast cancer biology. Gut microorganisms influence Trp availability, produce bioactive metabolites, and modulate host metabolic enzymes, thereby shaping immune and hormonal features of the breast tumor microenvironment. Microbiota-derived indole compounds and host-derived Kyn activate AhR-dependent signaling pathways that are associated with immunosuppression, tumor cell proliferation, invasion, and resistance to therapy. In parallel, interactions between microbial enzyme systems—including those involved in estrogen metabolism—and host metabolic pathways illustrate additional mechanisms through which the microbiota may affect disease progression. Together, these findings provide insight into metabolic reprogramming in breast cancer and may support future studies aimed at biomarker discovery and therapeutic development.

**Figure 5:**
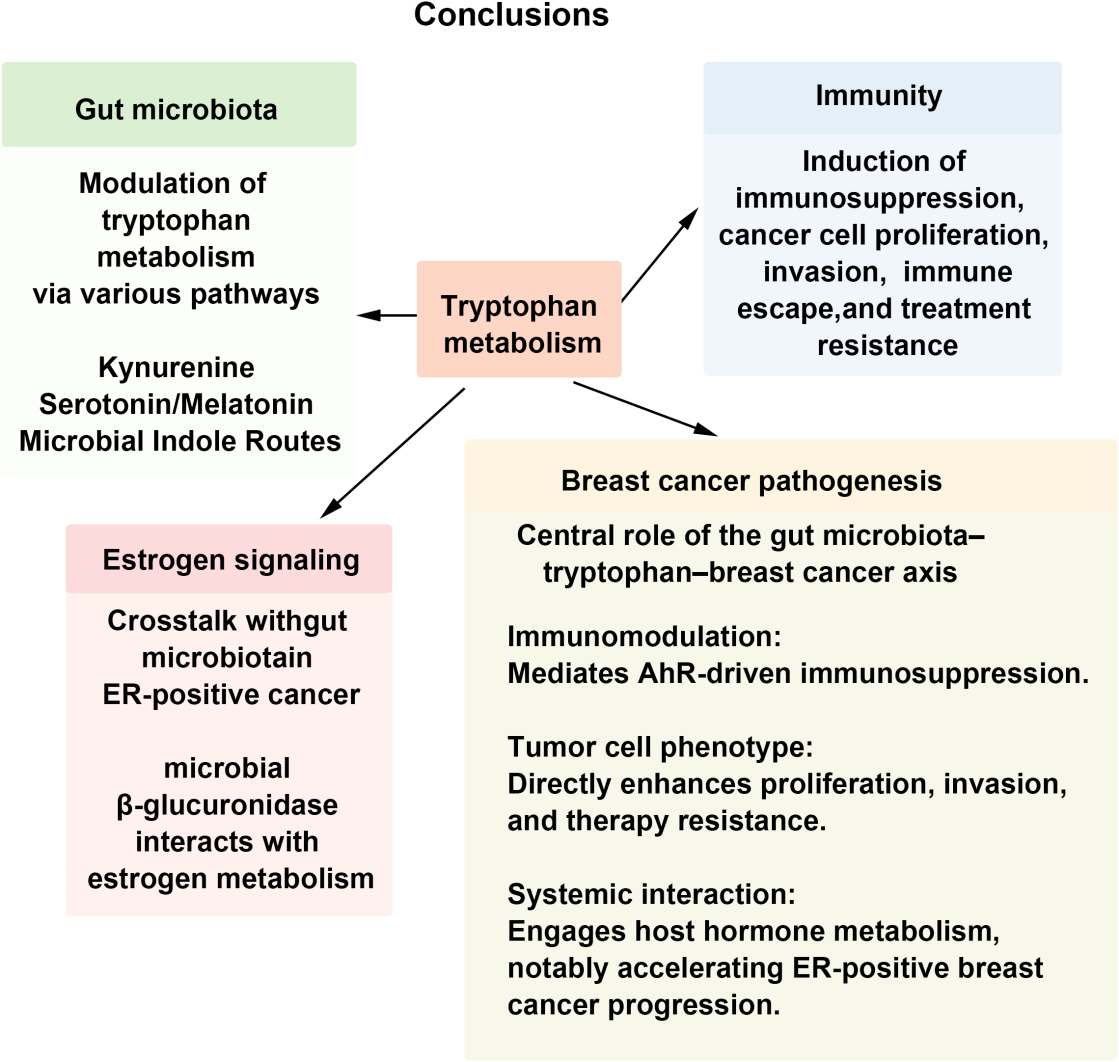
The gut microbiota-Trp metabolism-breast cancer axis serves as a key regulator of tumor behavior. Gut microbes shape the immune and hormonal landscape of the mammary TME by modulating Trp availability, generating bioactive metabolites, and regulating host metabolic enzymes. Microbial indole derivatives and host-derived Kyn activate the AhR pathway, thereby driving immunosuppression, cancer cell proliferation, and treatment resistance.

Despite recent progress, substantial gaps remain in this field. Much of the current mechanistic understanding is derived from germ-free or antibiotic-treated animal models, whereas human studies largely remain associative and are limited in their ability to establish causal relationships. From a technical perspective, challenges include the reliable detection of low-abundance metabolites, the marked heterogeneity across breast cancer subtypes, and interindividual variability in host-microbiota interactions, all of which complicate mechanistic interpretation. Future research should prioritize the establishment of longitudinal human cohorts, systematic integration of multi-omics data, and the development of targeted intervention strategies, including optimized probiotic formulations, engineered microbial therapies, and metabolite-based treatment approaches. In parallel, several critical questions remain to be addressed, such as defining metabolite thresholds relevant to breast cancer development, characterizing microbiota-metabolite heterogeneity across molecular subtypes, and translating microbial and metabolic features into clinically applicable stratified biomarkers. Progress in clinical translation will depend on rigorously designed trials and the development of patient stratification frameworks based on microbial and metabolic characteristics. Advancing these directions may ultimately support more precise prevention and treatment strategies targeting the microbiota-metabolism axis. The key concepts discussed in this review are consolidated in the **Figure 5**, which highlights the major mechanistic and translational themes.
